# Association between myocardial extracellular volume and strain analysis through cardiovascular magnetic resonance with histological myocardial fibrosis in patients awaiting heart transplantation

**DOI:** 10.1186/s12968-018-0445-z

**Published:** 2018-04-23

**Authors:** Yue Cui, Yukun Cao, Jing Song, Nianguo Dong, Xiangchuang Kong, Jing Wang, Yating Yuan, Xiaolei Zhu, Xu Yan, Andreas Greiser, Heshui Shi, Ping Han

**Affiliations:** 1 Department of Radiology, Union Hospital, Tongji Medical College, Huazhong University of Science and Technology, Wuhan 430022, China; 2 Department of Cardiovascular Surgery, Union Hospital, Tongji Medical College, Huazhong University of Science and Technology, Wuhan 430022, China; 3 MR Scientific NE Asia, Siemens Healthineers, Guangzhou, China; 4 MR Collaboration NE Asia, Siemens Healthineers, Shanghai, China; 5 Siemens Healthineers, Erlangen, Germany

**Keywords:** Myocardial fibrosis, Extracellular volume, Myocardial systolic strain, Collagen volume fraction

## Abstract

**Background:**

Cardiovascular magnetic resonance (CMR)-derived extracellular volume (ECV) and tissue tracking strain analyses are proposed as non-invasive methods for quantifying myocardial fibrosis and deformation. This study sought (1) to histologically validate myocardial ECV against the collagen volume fraction (CVF) measured from tissue samples of patients undergoing heart transplantation and (2) to detect the correlations between myocardial systolic strain and the myocardial ECV and histological CVF in patients undergoing heart transplantation.

**Methods:**

A total of 12 dilated cardiomyopathy (DCM) and 10 ischaemic cardiomyopathy (ICM) patients underwent T1 mapping with the Modified Look Locker Inversion recovery (MOLLI) sequence, T2 mapping and ECV. Myocardial systolic strain, including left ventricular global longitudinal (GLS), circumferential (GCS) and radial strain (GRS), were quantified using CMR cine images with tissue tracking analysis software. Tissue samples were collected from each of 16 segments of the explanted hearts and were stained with picrosirius red for histological CVF quantification.

**Results:**

A strong relationship was observed between the global myocardial ECV and histological CVF in the DCM and ICM patients based on a per-patient analysis (*r* = 0.904 and *r* = 0.901, respectively, *p* <  0.001). In the linear mixed-effects regression analysis, ECV correlated well with the histological CVF in the DCM and ICM patients on a per-segment basis (*β* = 0.838 and *β* = 0.915, respectively, *p* <  0.001). In the multivariate linear regression analysis, histological CVF was the strongest independent determinant of ECV in the patients awaiting heart transplantation (standardised *β* = 0.860, *p* <  0.001). However, the T2 time, GLS, GCS and GRS showed no significant associations with ECV and CVF in the patients awaiting heart transplantation.

**Conclusions:**

ECV derived from CMR correlated well with histological CVF, indicating its potential as a non-invasive tool for the quantification of myocardial fibrosis. Additionally, impaired myocardial systolic strains were not associated with the ECV and CVF in the patients awaiting heart transplantation.

## Background

Myocardial fibrosis is a common feature and the pathological basis of a variety of heart diseases, regardless of aetiology [1–4]. Myocardial fibrosis also leads to myocardial stiffness and dysfunction, resulting in the progression of heart failure and adverse clinical outcomes [2–4]. However, myocardial fibrosis might be reversible and has been proposed as a potential therapeutic target and prognostic factor [5, 6]. Therefore, detecting and quantifying myocardial fibrosis play an important role in diagnostic, prevention and prognostic assessments of cardiac diseases.

Cardiovascular magnetic resonance (CMR) imaging is a reliable non-invasive imaging modality that is widely used to evaluate cardiac morphology, function and tissue characterisation. CMR with late gadolinium enhancement (LGE) is a well-established modality for detecting regional myocardial fibrosis associated with adverse cardiovascular outcomes [7–10]. However, LGE cannot quantify diffuse myocardial fibrosis due to the lack of a remote myocardium as a reference. Recently, CMR T1 mapping technique has emerged as a non-invasive modality for quantifying myocardial fibrosis by measuring myocardial extracellular volume (ECV) and native T1 time [11–21]. Nevertheless, previous studies have shown relatively high variability in native T1 time for quantifying myocardial fibrosis. Studies by Lee et al. [20] and Bull et al. [21] demonstrated that native T1 mapping correlated with diffuse myocardial fibrosis by biopsy in patients with aortic stenosis. In contrast, Ravenstein et al. [14] reported no significant correlation between native T1 times and histological myocardial fibrosis at 3T. Compared to native T1 mapping, myocardial ECV, as derived from myocardial and blood pre- and post-contrast T1 relaxation time changes, has been validated as a preferred method for measuring extracellular matrix expansion [12–19]. While the correlation between ECV and histological collagen volume fraction (CVF) has been validated using endomyocardial biopsy, so far only sparse data exist on whole-heart histological validation from explanted hearts in patients undergoing heart transplantation. Furthermore, the role of T2 mapping in the histological validation of ECV remains uncertain.

Additionally, myocardial deformation analysis can supply useful information for the evaluation of myocardial function, which is very important in the management of patients with heart failure [22–24]. CMR tagging is considered a reference standard for the assessment of myocardial strain [25]. However, additional acquisition sequences and time-consuming protocols have limited its clinical application. Recently, new CMR tissue tracking technology, which agrees well with CMR tagging, has allowed for the assessment of global and regional myocardial strain by tracking the endocardial and epicardial borders during cardiac cycles using cine images; this technology has a higher signal-to-noise ratio (SNR) and a lower investment of time [22, 23, 26]. Currently, the relationships between myocardial systolic strain and CMR-derived ECV and histological CVF remain to be explored.

Therefore, the purposes of this study were to examine the relationship between CMR-derived ECV and histological CVF measured from explanted hearts and to explore the role of T2 mapping in the histological validation of ECV. Additionally, we aimed to determine whether the alterations of myocardial systolic strain are associated with ECV and histological myocardial fibrosis in patients undergoing heart transplantation.

## Methods

### Study population

Between June 2016 and July 2017, 40 consecutive patients with dilated cardiomyopathy (DCM) or ischaemic cardiomyopathy (ICM) on the heart transplant waiting list were referred for CMR. Of the 40 patients, 5 patients with DCM were prohibited from CMR due to a pacemaker; 5 DCM and 4 ICM patients were unable to complete CMR because of difficulties with breath-holding; and 2 DCM and 2 ICM patients lacked CMR images because they underwent CMR examinations in other hospitals. Thus, a total of 12 DCM and 10 ICM patients undergoing electrocardiogram, echocardiography, invasive coronary angiography, CMR and heart transplantation were included in the present study. The DCM diagnosis was based on (1) the presence of left ventricular (LV) dilatation with an increased LV end-diastolic volume index (EDVI) by CMR; (2) systolic dysfunction with a reduced LV ejection fraction (LVEF) < 35% and symptomatic heart failure with a New York Heart Association (NYHA) functional class III or greater; and (3) the absence of coronary artery disease by coronary angiography or subendocardial LGE indicating previous myocardial infarction [27, 28]. For all the ICM patients, coronary angiography was performed to diagnose coronary artery disease and LV systolic dysfunction with an LVEF ≤35%. ICM was diagnosed based on patient clinical histories as well as electrocardiogram (ECG), echocardiography, CMR, cardiac positron emission tomography (PET), invasive x-ray coronary angiography and histological samples [29]. Fifteen age- and sex-matched healthy subjects who responded to advertisements were recruited to participate in this study. The inclusion criteria included no known history of cardiovascular diseases, hypertension or diabetes mellitus, normal electrocardiography, and normal cardiac morphology, function and tissue characterisation (without LGE) by CMR. The exclusion criteria for all the subjects included renal insufficiency with an enhanced glomerular filtration rate (eGFR) < 30 mL/min/1.73 m^2^, an allergy to the contrast materials, and contraindications to CMR, including severe claustrophobia and device implantation. This study was approved by the Ethics Committee of Tongji Medical College, Huazhong University of Science and Technology. Written informed consent was obtained from all the participants.

### CMR imaging protocol

All the subjects underwent standard CMR examinations with a 1.5T scanner (MAGNETOM Aera, Siemens Healthineers, Erlangen, Germany). The cine images included the acquisition of three long-axis slices (two-, three-, and four-chamber) and a stack of short-axis slices covering the entire LV using a balanced steady state free precession (bSSFP) sequence. The cine image parameters were as follows: repetition time (TR)/echo time (TE) of 2.9/1.2 ms, slice thickness of 6 mm, field-of-view (FOV) of 360 × 270 mm^2^, matrix of 186× 256 pixels and flip angle of 80°. T1 mapping was performed on three standard LV short-axis slices (apex, mid, and basic ventricular levels) before and 15 min after the administration of a bolus of gadopentetate dimeglumine contrast agent (0.2 mmol/kg, Magnevist, Bayer Healthcare, Berlin, Germany) using a modified Look Locker inversion recovery (MOLLI) sequence with a 5(3)3 sampling scheme. The following typical MOLLI sequence parameters were used: TR/TE of 3.8/1.1 ms, slice thickness of 8 mm, FOV of 360 × 270 mm^2^, matrix of 144 × 256 pixels, voxel size of 1.3 × 1.3 × 8.0 mm^3^, flip angle of 35° and scan time of 11 heartbeats. T2 mapping was acquired in basal, mid and apical ventricular short-axis slices (identical to T1 mapping) before the contrast agent injection using a T2-prepared single-shot bSSFP sequence. The parameters were as follows: TR/TE of 3.8/1.3 ms, slice thickness of 8 mm, FOV of 360 × 270 mm^2^, matrix of 144 × 256 pixels and flip angle of 70°. A motion-correction algorithm was applied to correct the breathing and cardiac motion artefacts. LGE imaging of whole left ventricular short-axis slices and two-, three-, and four-chamber long-axis slices was performed 10 min after the intravenous injection of gadopentetate dimeglumine using a phase sensitive inversion recovery (PSIR) sequence. The LGE image parameters were as follows: TR/TE of 12.4/1.2 ms, slice thickness of 8 mm, FOV of 360 × 270 mm^2^, matrix of 256 × 192 pixels and flip angle of 40°.

### CMR image analysis

CMR images were analysed on a dedicated workstation using commercial software (Argus, Siemens Healthineers). Cardiac volumetric and functional parameters were quantified based on manual delineation of the endocardial and epicardial borders using a stack of continuous short-axis slice cine images (after excluding papillary muscles from the myocardium). All the parameters were indexed to the body surface area (BSA). The left ventricular EDVI, end-systolic volume index (ESVI), EF, stroke volume index (SVI), cardiac index and myocardial mass index were obtained automatically.

**Fig. 1:**
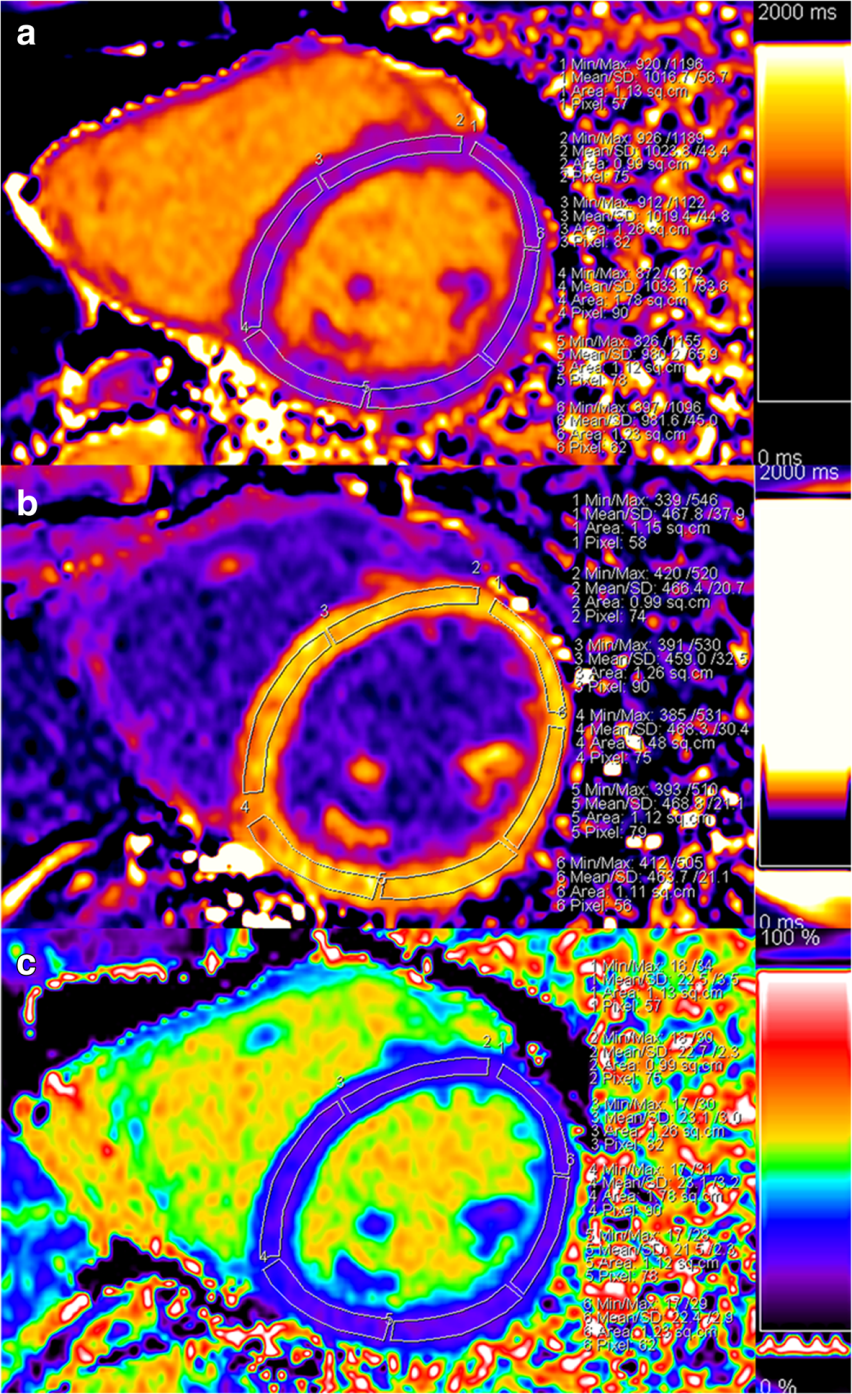
Example of cardiovascular magnetic resonance (CMR) T1 times and extracellular volume (ECV) maps measurements. CMR measurements of pre- (**a**) and post-contrast (**b**) T1 times and ECV (**c**) at the mid-ventricular short-axis level in a healthy subject

The haematocrit was obtained through a blood sample analysis on the day of the CMR scanning. The ECV maps were automatically calculated from pre- and post-contrast T1 times and haematocrit using a prototype inline processing function from Siemens. The myocardial T1, T2 times and ECV measurements were determined by drawing a region-of-interest (ROI) in each segment of each subject on a dedicated workstation with a ROI measuring tool (Siemens Healthineers, Erlangen, Germany), according to the 16-segment model from the American Heart Association (AHA) [30]. ROIs for all the subjects were drawn in a mid-wall region of the myocardium to minimise partial volume effects at the epicardial and endocardial borders. The ROIs were copied between the pre- and post-contrast T1, T2 and ECV maps. Segments with artefacts including poor breath holding, cardiac motion and off-resonance artefacts, as well as contamination from surrounding lung, liver, blood and epicardial fat, can lead to inaccurate T1 or ECV measurements and must be excluded. The image quality of the myocardial segments was visually divided into three levels—good, acceptable and poor—by two observers (YC and YKC), and discordant opinions were resolved by the third observer (HSS) to reach a consensus review [31]. The poor images were considered non-evaluable segments and were excluded from further analysis. The global myocardial T1, T2 and ECV values were calculated as an average of all evaluable segments for each subject. The method used to measure the T1 and ECV values is shown in Fig. 1. One observer measured the native T1 time and ECV and repeated the measurement after 4 weeks for intra-observer variability analysis. The other observer performed the measurement again, using the same method, for the inter-observer variability analysis.

The LGE was quantified using a threshold of 4 standard deviations (SD) above the mean signal intensity of the remote normal myocardium within the same slice [32]. The LGE images were assessed by an independent observer who was blinded to mapping and histological data. All the LV myocardium segments were classified as segments with and without LGE.

Myocardial deformation analysis was performed using dedicated tissue tracking software (CVI^42^, Circle, Calgary, Canada). Myocardial systolic strain analysis was quantified through manual delineation of the LV endocardial and epicardial borders in a stack of short-axis and three long-axis slice cine images with the initial contour place at end-diastole, as previously described [33]. The papillary muscles were excluded from the myocardium. The contours were manually corrected. The results of the LV GLS, GCS and GRS were automatically calculated and displayed for further analysis.

### Histological analysis

**Fig. 2:**
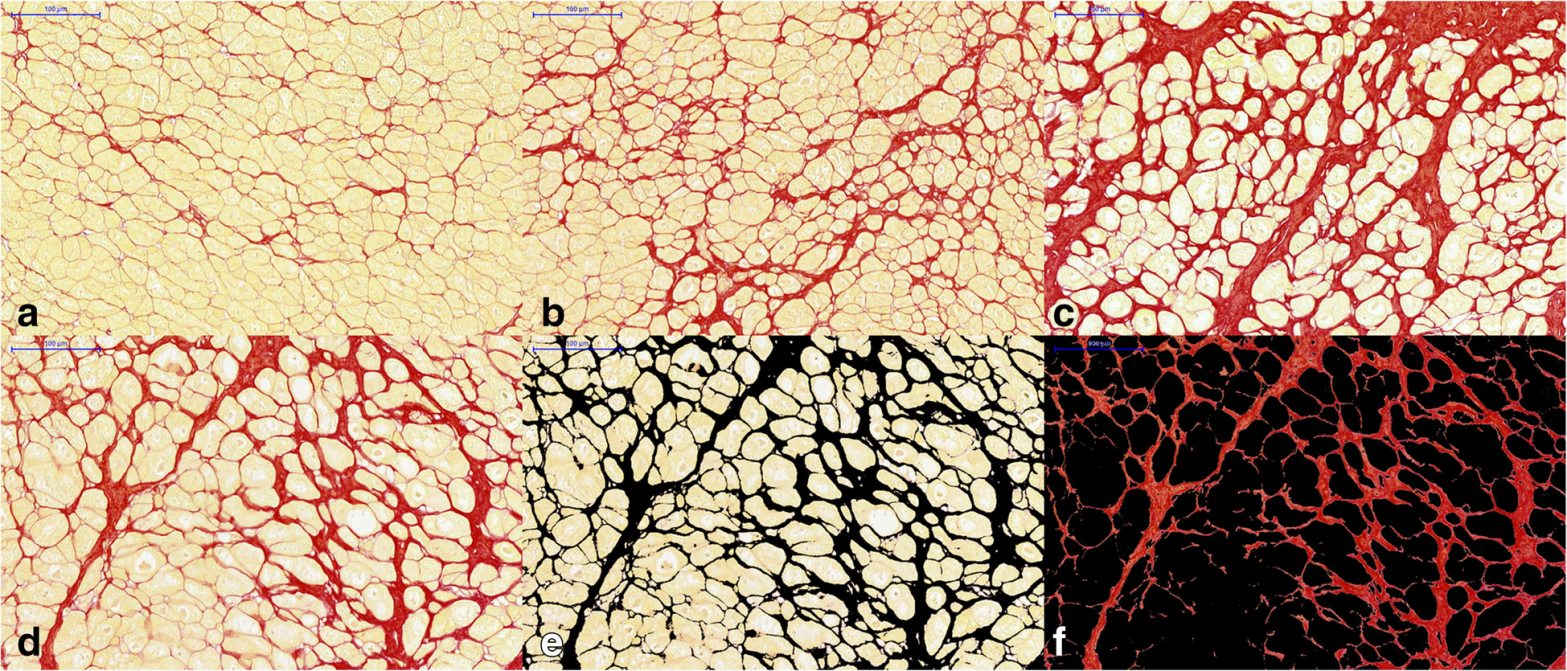
Representative histological analysis of patients undergoing heart transplantation. The whole-heart tissue samples were stained with picrosirius red (red = collagen, yellow = myocytes). Examples show mild (**a**, collage volume fraction (CVF) = 12.0%), moderate (**b**, CVF = 26.3%) and extensive fibrosis (**c**, CVF = 43.2%). The total myocardial area (**d**) and collagen area marked with black (**e**) were calculated semi-automatically using Image-Pro Plus software. Panel (**f**) shows the red collagen alone. The CVF was defined as the percentage of collagen area divided by the total myocardial area

After each patient underwent heart transplantation, the explanted hearts were cut in the apex, mid, and basic LV levels using the positions of CMR T1 mapping slices as the reference. Next, 16 tissue blocks were immediately taken from the LV apex, mid, and basic slices of each explanted heart; the positions of the tissue samples matched the sites of CMR T1 mapping of the 16 LV segments according to the AHA 16-segment model [30]. The tissue samples were immediately fixed with 10% buffered formalin, embedded in paraffin, and stained with picrosirius red. The stained sections were photographed at high-power (× 200) magnification after excluding artefacts and perivascular fibrosis tissues, as previously described [12]. Twelve high-power fields from each stained section were analysed using Image-Pro Plus 6.0 software (Media Cybernetics, Rockville, Maryland, USA). As shown in Fig. 2, the collagen was stained with red and myocytes with yellow. A colour-threshold macro-based calculation algorithm was used to separate collagen from myocardium. The collagen area was obtained from a combination of SD from mean signal and isodata automatic thresholding, as previously described [16, 17]. The histological CVF was defined as the percentage of collagen area divided by the total myocardial area. The average CVF of the 12 high-power fields was calculated as the myocardial fibrosis of each segment. All the tissue samples were analysed by an observer who was blinded to the CMR imaging results.

### Statistical analysis

Normality was detected using the Kolmogorov-Smirnov test. Continuous variables are presented as the mean ± SD, and categorical variables as percentages or frequencies. Comparisons between multiple groups were analysed using one-way ANOVA or the Kruskal-Wallis test, with the Bonferroni correction as the post hoc test, as appropriate. Categorical variables were analysed using the Chi-square test or the Fisher exact test. Correlation between ECV and CVF was assessed using Pearson’s or Spearman’s correlation coefficients, as appropriate. A linear mixed-effects regression analysis was used to assess the relationship between ECV and CVF for per-segment analyses. Univariate and multivariate linear regression analyses, with a stepwise algorithm, were performed to detect the determinants of ECV and CVF in the patients awaiting heart transplantation. Intra- and inter-observer variability of native T1 times, ECV and myocardial strain were assessed using an intra-class correlation coefficient (ICC) with 95% confidence intervals (CI). For all the tests, a two-sided *p* value < 0.05 was considered statistically significant. Statistical analyses were performed with IBM SPSS Statistics 21 (International Business Machines, Armonk, New York, USA), SAS® version 9.3 (SAS Institute Inc., Cary, North Carolina, USA) and GraphPad Prism 5.0 (GraphPad Software, San Diego, California, USA).

## Results

### Clinical characteristics of the study population

The baseline characteristics of the study population are listed in Table 1. The mean ages of the healthy subjects, DCM and ICM patients were similar (50.5 ± 6.5 vs. 49.3 ± 16.0 vs. 54.9 ± 7.3 years, *p* = 0.475). There were no significant differences in sex (86.7% vs. 91.7% vs. 90.0% males, *p* = 1.000), height, weight, body mass index (BMI), BSA and haematocrit. Heart rate was significant higher in the DCM patients than that in the healthy controls. The DCM and ICM patients showed a mean symptom duration of 6.5 and 2.2 years, respectively. The mean time between heart transplantation and CMR was 15 days (range: 0–32 days) and 27 days (range: 1–114 days) in patients with DCM and ICM, respectively.

**Table 1:** Demographics of the study population

Variables	Healthy subjects (*n* = 15)	DCM (*n* = 12)	ICM (*n* = 10)	*P* value
Age (years)	50.5 ± 6.5	49.3 ± 16.0	54.9 ± 7.3	0.475
Male (n, %)	13 (86.7)	11 (91.7)	9 (90.0)	1.000
Height (cm)	167.3 ± 4.4	167.4 ± 6.4	168.3 ± 4.6	0.889
Weight (kg)	68.5 ± 9.4	65.2 ± 18.8	66.5 ± 5.6	0.869
BMI (kg/m^2^)	24.4 ± 3.0	25.0 ± 3.7	23.1 ± 1.2	0.321
BSA (m^2^)	1.81 ± 0.14	1.77 ± 0.25	1.79 ± 0.07	0.967
Heart rate (bpm)	64 ± 7	83 ± 18*	75 ± 17	0.023
Haematocrit (%)	42.5 ± 3.8	41.9 ± 2.9	39.7 ± 6.6	0.675
Hypertension (n, %)	0 (0)	3 (25.0)	4 (40.0)	–
Diabetes mellitus (n, %)	0 (0)	4 (33.3)	4 (40.0)	–
Hyperlipidaemia (n, %)	0 (0)	1 (8.3)	2 (20.0)	–
Smoker (n, %)	6 (40.0)	4 (33.3)	7 (70.0)	0.263
Family history of coronary artery disease (n, %)	0 (0.0)	0 (0.0)	0 (0.0)	–
Duration (years)	–	6.5 ± 3.7	2.2 ± 1.5	–
Time between CMR and transplantation (days)	–	15 ± 11	27 ± 35	–
NYHA functional class III/IV (n, %)	–	2 (16.7)/10 (83.3)	1 (10.0)/9 (90.0)	–
NT-proBNP (pg/mL)	–	4680.9 ± 6064.7	3294.0 ± 3482.7	–
BUN (mmol/L)	–	8.6 ± 5.1	7.5 ± 3.0	–
Creatinine (μmol/L)	–	107.8 ± 51.5	125.8 ± 81.6	–

Values are expressed as the mean ± SD or n (%)

*p <  0.05 vs. controls

*DCM* Dilated cardiomyopathy, *ICM* Ischaemic cardiomyopathy, *BMI* Body mass index, *BSA* Body surface area, *CMR* Cardiac magnetic resonance, *NYHA* New York Heart Association, *NT-proBNP* N-terminal pro-brain natriuretic peptide, *BUN* Blood urea nitrogen

### CMR parameters comparison

Table 2 shows the CMR parameters of the study population. As expected, the DCM and ICM patients had significantly lower LVEF and greater EDVI, ESVI and myocardial mass index than the healthy controls (*p* <  0.05 for all). No significant differences in the LVSVI and cardiac index were observed between the three groups. Myocardial systolic strain analysis demonstrated that the LV GLS, GCS and GRS were lower in the DCM and ICM patients compared with those in the controls (*p* <  0.05 for all). Myocardial LGE was present in all the DCM and ICM patients. The mean native T1 time, T2 time and ECV were significantly higher in the DCM and ICM patients than those in the controls (*p* <  0.05 for all).

**Table 2:** CMR parameters and histological samples

Variables	Healthy subjects (n = 15)	DCM (n = 12)	ICM (n = 10)	*P* value
LV EF (%)	59.0 ± 5.3	13.1 ± 6.5*	18.0 ± 6.5*	< 0.001
LV EDVI (mL/m^2^)	67.7 ± 13.2	235.5 ± 67.0*	180.7 ± 36.2^#^	< 0.001
LV ESVI (mL/m^2^)	27.9 ± 7.6	204.6 ± 61.2*	149.1 ± 37.9^#^	< 0.001
LV SVI (ml/m^2^)	39.6 ± 7.1	30.3 ± 17.5	31.6 ± 12.1	0.088
LV cardiac index (L/min/m^2^)	2.5 ± 0.5	2.5 ± 1.5	2.4 ± 1.0	0.939
LV mass index (g/m^2^)	63.4 ± 7.8	127.9 ± 37.1*	124.9 ± 41.1*	< 0.001
LV GLS (%)	−16.2 ± 1.8	−4.4 ± 2.1*	− 4.3 ± 2.0*	< 0.001
LV GCS (%)	−18.2 ± 2.5	−4.7 ± 2.0*	− 5.1 ± 2.0*	< 0.001
LV GRS (%)	41.8 ± 9.4	7.9 ± 4.2*	8.1 ± 4.3*	< 0.001
Presence of LGE (n, %)	0 (0)	12 (100)	10 (100)	–
Native T1 time (ms)	1003 ± 19	1084 ± 60*	1073 ± 53^#^	< 0.001
ECV (%)	24.3 ± 1.7	35.0 ± 5.8*	37.8 ± 7.1*	< 0.001
CVF (%)	–	14.3 ± 4.6	17.0 ± 5.5	–
T2 mapping (ms)	45 ± 1	50 ± 3*	47 ± 2^#^	< 0.001

Values are expressed as the mean ± SD or n (%)

*p < 0.001 vs. controls; ^#^*p* < 0.05 vs. controls

*CMR* Cardiovascular magnetic resonance, *DCM* Dilated cardiomyopathy, *ICM* Ischaemic cardiomyopathy, *LV* Left ventricle, *EF* Ejection fraction, *EDVI* End-diastolic volume index, *ESVI* End-systolic volume index, *SVI* Stroke volume index, *GLS* Global longitudinal strain, *GCS* Global circumferential strain, *GRS* Global radial strain, *LGE* Late gadolinium enhancement, *ECV* Extracellular volume, *CVF* Collagen volume fraction

In the whole myocardium analysis, 11 of 192 (5.7%) myocardial segments in the DCM patients and 12 of 160 (7.5%) myocardial segments in the ICM patients were excluded due to artefacts identified by image quality assessment. In total, 64 segments with LGE and 117 segments without LGE from the DCM patients and 68 segments with LGE and 80 segments without LGE from the ICM patients were included in the analyses (Table 3). The mean native T1 time, T2 time and ECV of all segments and segments without LGE were significantly higher in the DCM and ICM patients compared with those in the controls (*p* <  0.001 for all). The ECV of all the segments from the ICM patients was significantly greater than that in the segments without LGE (*p* <  0.05).

**Table 3:** Comparison of the CMR and histological parameters between the healthy controls and patients based on segments

Variables	Healthy subjects	All segments	Segments without LGE	*P* value
DCM (n)	240	181	117	
Native T1 time (ms)	1003 ± 30	1081 ± 89*	1054 ± 55*	< 0.001
ECV (%)	24.3 ± 2.4	34.6 ± 7.3*	31.4 ± 4.0*	< 0.001
CVF (%)	–	14.0 ± 6.5	11.2 ± 3.9^#^	0.001
T2 mapping (ms)	45 ± 3	50 ± 6*	48 ± 4*	< 0.001
ICM (n)	240	148	80	
Native T1 time (ms)	1003 ± 30	1070 ± 93*	1076 ± 75*	< 0.001
ECV (%)	24.3 ± 2.4	37.4 ± 12.4*	30.2 ± 5.1*^#^	< 0.001
CVF (%)	–	16.8 ± 10.3	11.4 ± 4.4^#^	< 0.001
T2 mapping (ms)	45 ± 3	47 ± 5*	47 ± 4*	< 0.001

Values are expressed as the mean ± SD

*p < 0.001 vs. controls; ^#^*p* < 0.05 vs. all segments group

*DCM* Dilated cardiomyopathy, *ICM* Ischaemic cardiomyopathy, *CMR* Cardiovascular magnetic resonance, *LGE* Late gadolinium enhancement, *ECV* Extracellular volume, *CVF* Collagen volume fraction

### Histological validation

**Fig. 3:**
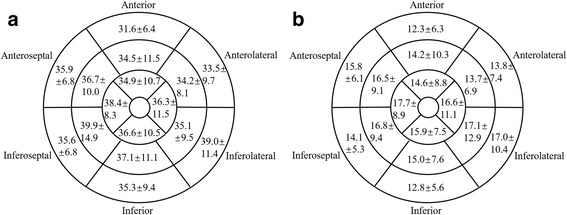
The bulls-eye plots show the ECV and CVF values. The mean ± SD (%) of left ventricular myocardial CMR-derived ECV (**a**) and histological CVF (**b**) was showed according to the AHA 16-segment model in the patients undergoing heart transplantation

**Fig. 4:**
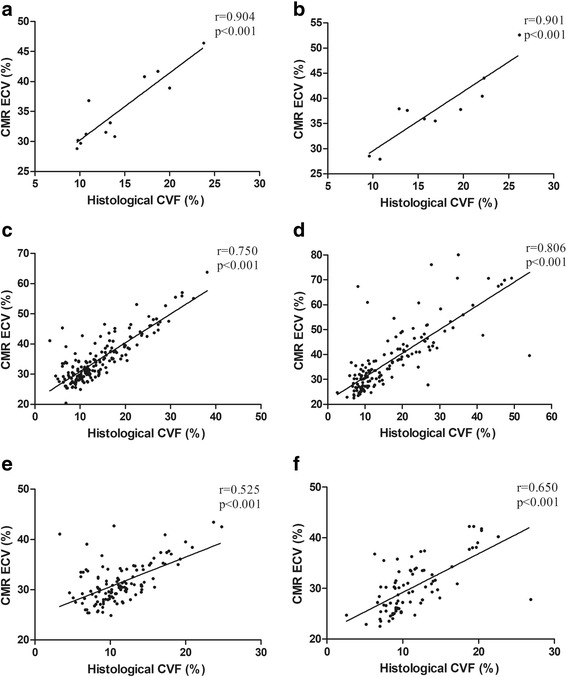
Correlations between the CMR-derived ECV and the histological CVF. Scatter plots show correlations between the myocardial ECV and the histological CVF in the DCM patients as follows: **a** based on a per-patient analysis, **c** based on a per-segment analysis containing all segments, and (**e**) based on a per-segment analysis excluding segments with LGE and in the ICM patients as follows: **b** based on a per-patient analysis, **d** based on a per-segment containing all segments, and **f** based on a per-segment analysis excluding segments with LGE

The mean histological CVF was 14.3 ± 4.6% (range: 9.7–23.8%) and 17.0 ± 5.5% (range: 9.6–26.2%) in the DCM and ICM patients, respectively. Figure 3 shows a segmental comparison of the LV myocardial CMR-derived ECV and histological CVF as the mean ± SD according to the AHA 16-segment model in patients awaiting heart transplantation. Based on the per-patient analysis, the ECV values strongly correlated with the histological CVF in the DCM and ICM patients (*r* = 0.904, *p* <  0.001 and *r* = 0.901, *p* <  0.001, respectively; Fig. 4). The per-segment analysis also showed that the ECV correlated well with the histological CVF in the DCM and ICM patients (*r* = 0.750, *p* <  0.001 and *r* = 0.806, *p* <  0.001, respectively; Fig. 4). After excluding the segments with LGE, the ECV was moderately correlated with the histological CVF in the DCM and ICM patients (*r* = 0.525, *p* <  0.001 and *r* = 0.650, *p* <  0.001, respectively; Fig. 4). The per-segment analysis using linear mixed-effects regression showed that there was a significant relationship between ECV and histological CVF in the DCM and ICM patients (*β* = 0.838, *p* <  0.001 and *β* = 0.915, *p* <  0.001, respectively).

Table 4 shows the results of the univariate and multivariate linear regression analyses of the ECV, CVF and other indices in the patients awaiting heart transplantation. In the univariate regression analysis, the ECV was associated with sex, N-terminal pro-brain natriuretic peptide (NT-proBNP), time between CMR and transplantation and histological CVF in patients awaiting heart transplantation. However, in the multivariate regression analysis, the independent determinants of ECV were sex and histological CVF (standardised *β* = 0.250, *p* = 0.007 and standardised *β* = 0.860, *p* <  0.001, respectively). In addition, the univariate regression analysis showed that histological CVF was correlated with the NT-proBNP, time between CMR and transplantation, native T1 time and ECV in the patients awaiting heart transplantation. The multivariate regression analysis demonstrated that the ECV was the independent determinant of the histological CVF (standardised *β* = 0.911, *p* <  0.001). However, no significant associations between ECV and histological CVF with left ventricular GLS, GCS, GRS and T2 mapping were observed in the patients awaiting heart transplantation (*p* > 0.05 for all).

**Table 4:** Univariate and multivariate regression analysis for ECV and CVF in the patients awaiting heart transplantation

	ECV				CVF			
	Univariate analysis		Multivariate analysis		Univariate analysis		Multivariate analysis	
Variables	*r* value	*P* value	Standardised *β*	*P* value	*r* value	*P* value	Standardised *β*	*P* value
Age (years)	−0.032	0.888			0.174	0.439		
Sex (%)	0.425	0.049	0.250	0.007	0.196	0.381		
BMI (kg/m^2^)	−0.130	0.563			−0.064	0.776		
Heart rate (bpm)	−0.199	0.374			0.269	0.227		
Haematocrit (%)	−0.400	0.065			−0.307	0.164		
Hypertension (%)	−0.030	0.896			0.152	0.500		
Diabetes mellitus (%)	−0.161	0.474			0.086	0.704		
Hyperlipidaemia (%)	−0.114	0.612			−0.014	0.951		
Smoker (%)	−0.370	0.090			−0.349	0.112		
Duration (years)	−0.174	0.440			−0.094	0.678		
Time between CMR and transplantation (days)	0.562	0.006		0.507	0.451	0.035		0.581
NT-proBNP (pg/mL)	0.602	0.005		0.701	0.549	0.012		0.995
LV EF (%)	−0.258	0.246			−0.126	0.576		
LV ESVI (mL/m^2^)	−0.180	0.422			−0.316	0.153		
LV EDVI (mL/m^2^)	−0.258	0.246			−0.262	0.240		
LV SVI (mL/m^2^)	−0.325	0.140			−0.244	0.275		
LV cardiac index (L/min/m^2^)	−0.359	0.101			−0.320	0.146		
LV mass index (g/m^2^)	−0.400	0.065			−0.234	0.295		
LV GLS (%)	0.313	0.168			0.316	0.163		
LV GCS (%)	0.381	0.088			0.282	0.215		
LV GRS (%)	−0.381	0.088			−0.290	0.202		
Native T1 time (ms)	–	–			0.508	0.016		0.138
ECV (%)	–	–			0.907	< 0.001	0.911	< 0.001
CVF (%)	0.907	< 0.001	0.860	< 0.001	–	–		
T2 mapping (ms)	0.339	0.143			0.258	0.272		

*CMR* Cardiovascular magnetic resonance, *BMI* Body mass index, *NT-proBNP* N-terminal pro-brain natriuretic peptide, *LV* Left ventricle, *EF* Ejection fraction, *ESVI* End-systolic volume index, *EDVI* End-diastolic volume index, *SVI* Stroke volume index, *GLS* Global longitudinal strain, *GCS* Global circumferential strain, *GRS* Global radial strain, *ECV* Extracellular volume, *CVF* Collagen volume fraction

### Repeatability analysis

The intra-observer and inter-observer variability were analysed for the 15 healthy subjects. The ICCs and 95% CIs for intra-observer and inter-observer agreement were 0.974 (95% CI: 0.925, 0.991) and 0.967 (95% CI: 0.904, 0.989) for the ECV measurements and 0.942 (95% CI: 0.836, 0.980) and 0.922 (95% CI: 0.785, 0.973) for the native T1 times measurements, respectively. The ICCs and 95% CIs for intra-observer and inter-observer agreement were 0.882 (95% CI: 0.685, 0.959) and 0.894 (95% CI: 0.714, 0.963) for GLS, 0.964 (95% CI: 0.897, 0.988) and 0.911 (95% CI: 0.757, 0.969) for GCS, and 0.942 (95% CI: 0.836, 0.980) and 0.921 (95% CI: 0.781, 0.973) for GRS, respectively.

## Discussion

The results of this study demonstrated that (1) myocardial ECV calculated by CMR T1 mapping correlated well with the degree of myocardial fibrosis measured in whole-heart histological samples from the patients undergoing heart transplantation; (2) T2 mapping was increased in the patients awaiting heart transplantation but was not related to myocardial ECV and histological CVF after adjusting for potential confounding factors in the multivariate regression analysis; and (3) in this cohort of patients, the LV GLS, GCS and GRS were decreased, and impaired myocardial systolic strain was not associated with CMR-derived ECV and histological myocardial fibrosis.

CMR T1 mapping is increasingly being recommended as a non-invasive diagnostic tool for myocardial tissue characterisation. Previous studies have validated the use of CMR T1 times and myocardial ECV against biopsy samples in patients with severe aortic stenosis or regurgitation, DCM, hypertrophic cardiomyopathy and ICM [12, 14–16, 34]. Although the different field strengths and CMR T1 mapping techniques limit comparability, previous studies have generally indicated that accurate measurements of ECV calculated by CMR T1 mapping reflected actual myocardial fibrosis or extracellular matrix expansion in patients with a variety of cardiac diseases [12–18]. Our results are consistent with these studies, and we comprehensively demonstrated good correlations between whole-heart ECV measurements and histological myocardial fibrosis for 22 patients awaiting heart transplantation. To the best of our knowledge, most previous studies have evaluated myocardial fibrosis using an endomyocardial biopsy as the reference standard. Myocardial samples by biopsy can only reflect a few millimetres of subendocardial pathological information, which may be affected by procedure-related tissue distortion [35]. Sampling-induced contraction bands can dislocate intracellular organelles and alter the structural relationship between myocytes and the extracellular matrix [35]. Furthermore, if an endomyocardial biopsy is performed from the right ventricular side of the interventricular septum, the pathological data will not necessarily reflect LV information. However, the above limitations do not exist in the whole-heart histological samples from explanted hearts in this study. Additionally, for endomyocardial biopsy, it is impossible to ensure that samples correspond exactly to the CMR imaging sites, and they might not necessarily be representative of whole-heart myocardial fibrosis. However, our tissue samples were collected from each of 16 segments of the explanted hearts, which might better correspond to the site of CMR T1 mapping and could more accurately provide whole-heart histological validation.

Additionally, previous studies by Miller et al. and Iles et al. have validated CMR T1 mapping against histological samples from patients using 6 and 11 explanted hearts, respectively [12, 34]. However, the study by Iles et al. only analysed post-contrast T1 times against histological CVF without ECV measurements in a single mid-ventricular short-axis slice, and thus their results can only partially assess myocardial fibrosis due to the influences of renal excretion [34]. ECV corrected by the haematocrit minimises the impact of some of confounding factors compared with T1 times and can provide accurate information for the quantification of myocardial fibrosis. In addition, their analysis considered a limited number of patients with a variety of heart disease aetiologies, including DCM, ICM, and restrictive and congenital heart diseases, which might make their results less useful. In our study, we analysed 12 DCM and 10 ICM patients separately and demonstrated good correlations between myocardial ECV and histological CVF, as measured by whole-heart histological samples from these patients. ECV measurements can be an effective alternative for clinical risk stratification and the prognostic evaluation of antifibrotic treatment. Furthermore, we analysed CMR T1, T2 mapping, ECV and myocardial systolic strain in one-stop examinations, without extra images, which provided comprehensive insight into myocardial fibrosis, oedema and cardiac function.

Similar to CMR T1 mapping, T2 mapping techniques can also be used to assess myocardial tissue properties. It has been suggested that CMR T2 mapping can be used to detect myocardial oedema in acute myocardial infarction, myocarditis or cardiac allograft rejection [36–38]. In the present study, we enrolled a group of end-stage heart failure patients undergoing heart transplantation, and myocardial inflammation might have occurred in these patients. Our results suggest that the T2 times in the DCM and ICM patients were slightly increased compared with those in the healthy subjects. However, in the multivariate regression analysis, the T2 time did not significantly contribute to the ECV measurement, which indicated that myocardial oedema might play a minor role in the expansion of the extracellular space in this study cohort and was unlikely to alter the ECV value. A previous study by Bohnen et al. reported that the optimal cut-off value of the global myocardial T2 time was 60 ms for active myocarditis in a 1.5-T scanner [36]. The T2 value in the present study was lower than the above cut-off value, which suggested that the pathological changes in the study patients were primarily myocardial fibrosis, without obvious myocardial oedema. Therefore, the good correlations between ECV and histological CVF observed in our study demonstrated that CMR-derived ECV is a useful tool for the quantification of myocardial fibrosis.

Recently, an advanced CMR tissue tracking technique was proposed as a non-invasive and accurate modality for myocardial deformation analysis using cine images. Myocardial systolic strains can be used to characterise early myocardial dysfunction in clinical practice [22]. In the present study, the decreased GLS, GCS and GRS in the patients awaiting heart transplantation suggested serious LV myocardial dysfunction. In the multivariate regression analysis, GLS, GCS and GRS were not associated with ECV and histological myocardial fibrosis in patients awaiting heart transplantation. Previous studies have shown relatively high variability in the relationship between myocardial fibrosis and myocardial systolic strains [39, 40]. A recent study by Cameli et al. indicated that GLS was associated with the degree of myocardial fibrosis by tissue samples in patients requiring heart transplantation [39]. However, Dusenbery et al. reported that decreased GLS correlated with LGE but not ECV [40]. Our results also showed that decreased LV GLS, GCS and GRS showed no correlation with histological fibrosis or ECV in the patients undergoing heart transplantation. The differences between various studies might be associated with clinical stage, duration, myocardial strain acquisition method, pathogenesis and medical treatment in different diseases. We studied a group of patients with end-stage heart diseases. The pathogenesis and pathological processes are highly complicated and diverse. Myocardial fibrosis could be just one of the many causes of impaired myocardial systolic strain in the study patients. Therefore, further multi-centre studies with larger sample sizes are required to validate these results.

### Study limitations

Our study evaluated the correlation between ECV and histological CVF using whole-heart tissue samples from 22 patients awaiting heart transplantation. Although the number of patients was limited, the sample sizes were relatively large, given that we used whole-heart tissue samples. Furthermore, we aim to collect more whole-heart tissue samples from patients awaiting heart transplantation at our institution for further study. The time delay between CMR and heart transplantation was a major factor affecting the results. However, the mean time between transplantation and CMR was less than one month in our study, which would not allow for a significant change in the myocardial collagen content [34]. Additionally, in the multivariate analysis, the time between CMR and transplantation was not associated with histological myocardial fibrosis. Although this study validated ECV against histological CVF in whole-heart samples, the tissue samples represented only small myocardial sections, which cannot be accurately located using CMR. Thus, sampling bias still existed. However, this technique is more robust than endomyocardial biopy, which only reflects the subendocardial part of the myocardium and not the whole myocardium. Finally, excluding patients with pacemakers and with difficulty about breath-holding may induce a selection bias.

## Conclusions

Our results show that CMR-derived ECV correlates well with the histological CVF, indicating its potential use as a novel non-invasive imaging technique for quantifying myocardial fibrosis and for guiding clinical interventions and monitoring clinical therapy. Decreased LV myocardial systolic strain was not related to histological myocardial fibrosis or ECV in the present study.

## Abbreviations

AHA: American Heart Association
ANOVA: Analysis of variance
BSA: Body surface area
bSSFP: Balanced steady state free precession
CMR: Cardiovascular magnetic resonance
CVF: Collagen volume fraction
DCM: Dilated cardiomyopathy
ECG: Electrocardiogram
ECV: Extracellular volume
EDVI: End-diastolic volume index
eGFR: Estimated glomerular filtration rate
ESVI: End-systolic volume index
FOV: Field-of-view
ICM: Ischaemic cardiomyopathy
LGE: Late gadolinium enhancement
LV: Left ventricle/left ventricular
LVEF: Left ventricular ejection fraction
MOLLI: Modified Look Locker Inversion recovery
NYHA: New York Heart Association
PSIR: Phase sensitive inversion recovery
ROI: Region-of-interest
SNR: Signal-to-noise ratio; bSSFP: Balanced steady state free precession
SVI: Stroke volume index
TE: Echo time
TR: Repetition time
